# 21st century TeleWEAR: intergenerational and co-productive design of Telecare products

**Published:** 2012-06-15

**Authors:** Lorna Gail Bernard, Andrea F Taylor

**Affiliations:** Moray Community Health and Social Care Partnership, UK; Glasgow School of Art, UK

**Keywords:** co design, pendant, collaboration

## Abstract

Telecare products and services are fast becoming important features in the delivery of public services as they strive to deliver health and social care services in home settings rather to traditional models of institutionalised care. This methodology concurs with service-users’ desire to remain at home in independence for as long as possible. The aesthetic design of perhaps the most iconic piece of Telecare equipment—the red-button pendant has changed little in around 50 years. Anecdotal evidence supports the view that it is unattractive—particularly to younger users—and promotes a degree of stigmatisation. This project therefore brought together service users, potential service users, suppliers and young design students to work together to innovate the current pendant trigger design.

The project was based around the concepts of co-production and intergenerational practice to achieve true innovation. Evaluation of the project has been shared with leading manufacturers who have acknowledged the beneficial outcomes it produced by involving users as consumers in design. Participants reported positive personal outcomes such as increased motivation and self esteem. As a result we now have an ongoing Telehealthcare Involvement Group in Moray, with whom manufacturers are still engaged.

